# Cardiovascular Disease Risk Factors Among Children and Adolescents With Depression

**DOI:** 10.3389/fpsyt.2021.702737

**Published:** 2021-08-13

**Authors:** Daphne J. Korczak, Kristin Cleverley, Catherine S. Birken, Tony Pignatiello, Farid H. Mahmud, Brian W. McCrindle

**Affiliations:** ^1^Department of Psychiatry, Hospital for Sick Children, Toronto, ON, Canada; ^2^Research Institute, Hospital for Sick Children, Toronto, ON, Canada; ^3^Department of Psychiatry, Faculty of Medicine, University of Toronto, Toronto, ON, Canada; ^4^The Margaret and Wallace McCain Centre for Child, Youth and Family Mental Health, Centre for Addiction and Mental Health, Toronto, ON, Canada; ^5^Department of Psychiatry, Faculty of Medicine, University of Toronto, Toronto, ON, Canada; ^6^Lawrence S. Bloomberg Faculty of Nursing and Department of Psychiatry, University of Toronto, Toronto, ON, Canada; ^7^Child Health Evaluative Sciences, The Hospital for Sick Children, Toronto, ON, Canada; ^8^Department of Paediatrics, Faculty of Medicine, University of Toronto, Toronto, ON, Canada; ^9^Division of Endocrinology, Hospital for Sick Children, Toronto, ON, Canada; ^10^Division of Paediatric Medicine, Hospital for Sick Children, Toronto, ON, Canada; ^11^The Labatt Family Heart Centre, Hospital for Sick Children, Toronto, ON, Canada

**Keywords:** child and adolescent psychiatry, major depressive disorder, obesity, hyperlipidemia, hypertension, early intervention, cardiovascular disease risk factors

## Abstract

**Aim:** To examine CVD risk factors among children and adolescents with Major Depressive Disorder (MDD).

**Methods:** A cross-sectional study of 77 children and adolescents (mean age 14.1 years, 74% female) referred to a pediatric depression program. MDD was assessed using a semi-structured diagnostic interview. Cardiovascular assessments included family cardiovascular disease (CVD) history, cigarette smoking, body mass index (BMI), blood pressure, lipid and glucose concentrations. CVD risk factors among healthy weight and overweight/obese participants were compared.

**Results:** Forty-six percent of participants had a family history of early CVD. On examination, 25% of participants had a BMI in overweight/obese range, and 25% of children had pre-hypertension (14%) or hypertension (11%). Total cholesterol levels were elevated among 28% of participants. Overweight/obese participants had increased non-HDL cholesterol concentrations compared with healthy-weight participants (36 vs. 10%, *p* = 0.01). There were no significant differences between healthy and overweight/obese groups for other CVD risk factors, including HDL cholesterol concentration, plasma glucose concentration, hypertension, cigarette smoking, and family history of early CVD. More than half (52%) of participants had at least two CVD risk factors.

**Conclusion:** CVD risk factors are prevalent among children and adolescents with MDD. Routine CVD risk factor screening may be warranted among MDD youth, regardless of BMI, and may provide a valuable opportunity for prevention of future CVD.

## Introduction

Cardiovascular disease (CVD) and Major Depressive Disorder (MDD) are two significant public health problems that are major contributors to the global burden of mortality and morbidity (1–3). In addition, MDD is an independent predictor of CVD, associated with increased myocardial infarction (OR = 1.6; 95% CI 1.34–1.92) (4) and risk of CVD-related death (standardized mortality ratio 1.5–1.9) among affected individuals (5, 6). Studies have further confirmed that individuals with MDD are at increased risk of poor CVD outcomes even after controlling for their shared comorbidities (e.g., smoking) and other key covariates (e.g., obesity, anti-depressant medication use) (7).

Although most research to date has been conducted in middle-aged and older adults, several prospective epidemiologic studies have reported that the association between MDD and CVD begins decades prior to clinically manifest disease, confirming that youth with MDD are at increased risk of premature atherosclerosis and early CVD-related mortality compared with their non-depressed peers (8). Moreover, studies among youth have also focused primarily on single factors or lifestyle choices (e.g., sedentary/physical activity). Indeed, increased sedentary behavior and/or decreased physical activity among people with MDD leading to overweight and obesity is one suggested mechanism by which depression may predispose an individual to CVD (9, 10).

To our knowledge, only one study has examined clinically pertinent physiological markers of CVD risk among adolescents with MDD. Waloszek et al. (11) reported an increase in a cumulative CVD risk score among 17 youth with MDD in the community. In addition to the small sample size, the study was limited in the clinical appraisal of risk factor score components, in that whether specific risk score components (e.g., lipoproteins) were above clinical thresholds was not identified. As such, more precise inspection of the specific risk factors which may underlie the association, necessary for directing future research, could not be made. The objectives of the present study were to examine the degree to which children with MDD manifest clinical signs and symptoms that are known to be risk factors for CVD, and the extent to which elevated physiologic markers differ among healthy- and overweight groups.

## Methods

### Participants

Participants were recruited from a child and adolescent psychiatry clinic for youth with MDD at The Hospital for Sick Children (SickKids), a tertiary care children's hospital in downtown Toronto, Canada. Participants were boys and girls under the age of 18 years with current or recent MDD as defined by the Diagnostic and Statistical Manual for Mental Disorders 5th edition (DSM-5) (12) and at least one parent available to participate. Exclusion criteria included inability to provide informed consent/assent (e.g., psychotic disorder, developmental delay), history of hypomania/mania and significant chronic medical illness (e.g., rheumatologic disease, cancer).

Participants were referred to the study via the Children's Integrated Mood and Body (CLIMB) program, an embedded outpatient clinical research program within the Department of Psychiatry. Youth with relevant reasons for referral (e.g., sadness, decreased motivation) and increased self-reported depressive symptoms (see measures below) are referred to CLIMB via the departmental centralized intake system, which receives referrals from a wide variety of clinicians and settings, including family physicians, pediatricians, nurse practitioners, emergency medicine clinicians, and psychiatrists. Assessments for MDD and CVD risk factors in the present study occurred following initial referral for psychiatric evaluation. Participants and their parent/guardian provided written informed consent. This study was approved by the SickKids' Research Ethics Board.

### Clinical Procedure and Measures

Psychiatric diagnoses: Current and lifetime diagnoses were determined by standardized semi-structured psychiatric interview using the Schedule for Affective Disorders and Schizophrenia for School-Age Children, Present and Lifetime Version (KSADS-PL) (13). The KSADS DEP-C was used to assess depressive symptoms in current and previous depressive episodes for confirmation of MDD diagnosis. Youth and parent each separately served as informants with information incorporated from each informant. All interviewers had completed a master's degree in a health science field with experience administering standardized semi-structured psychiatric interviews and were trained in the KSADS-PL. After the interview was completed, consensus conferences were held with a child-adolescent psychiatrist (D.K.) blinded to research participation status for diagnostic confirmation. Youth also completed the Centre for Epidemiologic Studies of Depression in Children (CES-DC), (14) a reliable 20 item self-report measure of depressive symptoms that has been validated among children ages 8 to 18 years, with higher scores indicating greater symptom burden. A threshold score of 25 on the CES-DC was used for referral to the CLIMB program, consistent with research reporting the need for employment of higher thresholds in clinical vs. community samples (15, 16).

Cardiovascular Risk Factors: Height and weight were measured in light clothing to the nearest 0.1 cm and 0.1 kg, respectively, using a Health o meter Professional stadiometer (Sunbeam Products, Inc). Body Mass Index (BMI) was computed as weight divided by height squared (kg/m2) and BMI percentile was calculated using recommended age- and sex-based standardized norms (17). Overweight was defined as BMI greater than the 85th and less than the 95th percentile; obese was defined as BMI equal to or greater than the 95th percentile, in keeping with recommendations of the National Institute of Health Expert Panel on Integrated Guidelines for Cardiovascular Health and Risk Reduction in Children and Adolescents (NIH-EP-CVH) (18). Systolic and diastolic blood pressure (BP) was measured using a DINAMAP ProCare DCP101X-CE automated blood pressure monitor (GE Healthcare) with the participant resting in the seated position for a minimum of 5 min and repeated following a further 5 min period, with measures averaged across readings and repeated a third time if discrepancies >10 mmHg in the first two readings were present. Blood pressure percentile was calculated based on age, sex and height based norms (19). BP less than the 90th percentile was considered healthy; prehypertension was defined as BP at or greater than the 90the and less than the 95th percentile; and hypertension was defined as being at or greater than the 95th percentile (18). Fasting plasma lipid (total cholesterol, low density lipoprotein [LDL] cholesterol, high density lipoprotein [HDL] cholesterol, triglyceride [TG]) and glucose concentrations were classified as being acceptable (healthy), borderline-high, or high as defined by the NIH-EP-CVH (18). Family history of early CVD was assessed using an interviewer administered family history inventory (with parents) to identify the cardiovascular status of first and second degree relatives, depending on parental age. Family history of early CVD was defined as history of a parent and/or sibling with a history of treated angina, myocardial infarction, percutaneous coronary catheter interventional procedure, coronary artery bypass grafting, stroke or sudden cardiac death prior to the age of 65 years among participants' mothers or 55 years among participants' fathers (18). In the event that parents of participants had not reached these age thresholds, the cardiovascular health history of second degree relatives was considered using the same definitions. Presence of cigarette smoking was defined as smoking within the last 30 days, and determined by self-administered questionnaire regarding youth lifestyle and behaviors.

### Data Analysis

Participant data collected from July 2018 to March 2020 were included. This is a descriptive study in which data were summarized using means and standard deviations for continuous variables and counts/percentages for nominal variables. Presence of CVD risk factors was determined using thresholds from the NIH-EP-CVH as defined above. To compare individual CV risk factor levels between non-obese and obese groups, student's *t*-tests and chi-square tests were used for continuous and categorical data, respectively. Analyses were undertaken using IBM SPSS Statistics for Windows, version 23 (IBM Corp., Armonk, N.Y., USA)(20).

## Results

### Demographic and Clinical Characteristics

Demographic and clinical characteristics of the 77 participants are summarized in Table 1. There were more girls than boys (74 vs. 26%) in the sample, which is typical of adolescent depression (21). Participants were an average of 14.1 (SD 2.4) years of age and had experienced clinically significant depressive symptoms for a mean of 2.4 years at the time of study recruitment. Participants reported experiencing a high level of depressive symptom burden. At the time of study participation, a minority of participants (27%) reported current antidepressant medication use. The majority of the sample (72%) was Caucasian, with the predominant minority groups of either Asian or mixed ethnic background. The majority of parents of participants had completed high school and had attended some type of post-secondary education program, including college, university, or technical program.

**Table 1:** Participant demographic and depression characteristics.

	***n* (%)**	***N***
Sex (female)	57 (74)	77
Ethnicity (child)		65
Caucasian	47 (65)	
Asian	6 (8)	
Mixed	10 (13)	
Other	2 (3)	
Maternal education		63
Post-secondary education	57 (74)	
Paternal education		63
Post-secondary education	48 (62)	
Current antidepressant medication use	21 (27)	77
	**Mean (SD)**	***N***
Age (years)	14.1 (2.4)	77
Age at depression onset (years)	12.4 (2.4)	75
Duration of depressive symptoms (years)	2.4 (2.1)	75
CES-DC^*^ score	42.3 (9.5)	70

* *CES-DC, Centre for epidemiologic studies of depression in children*.

### CVD Risk Factors

**Figure 1:**
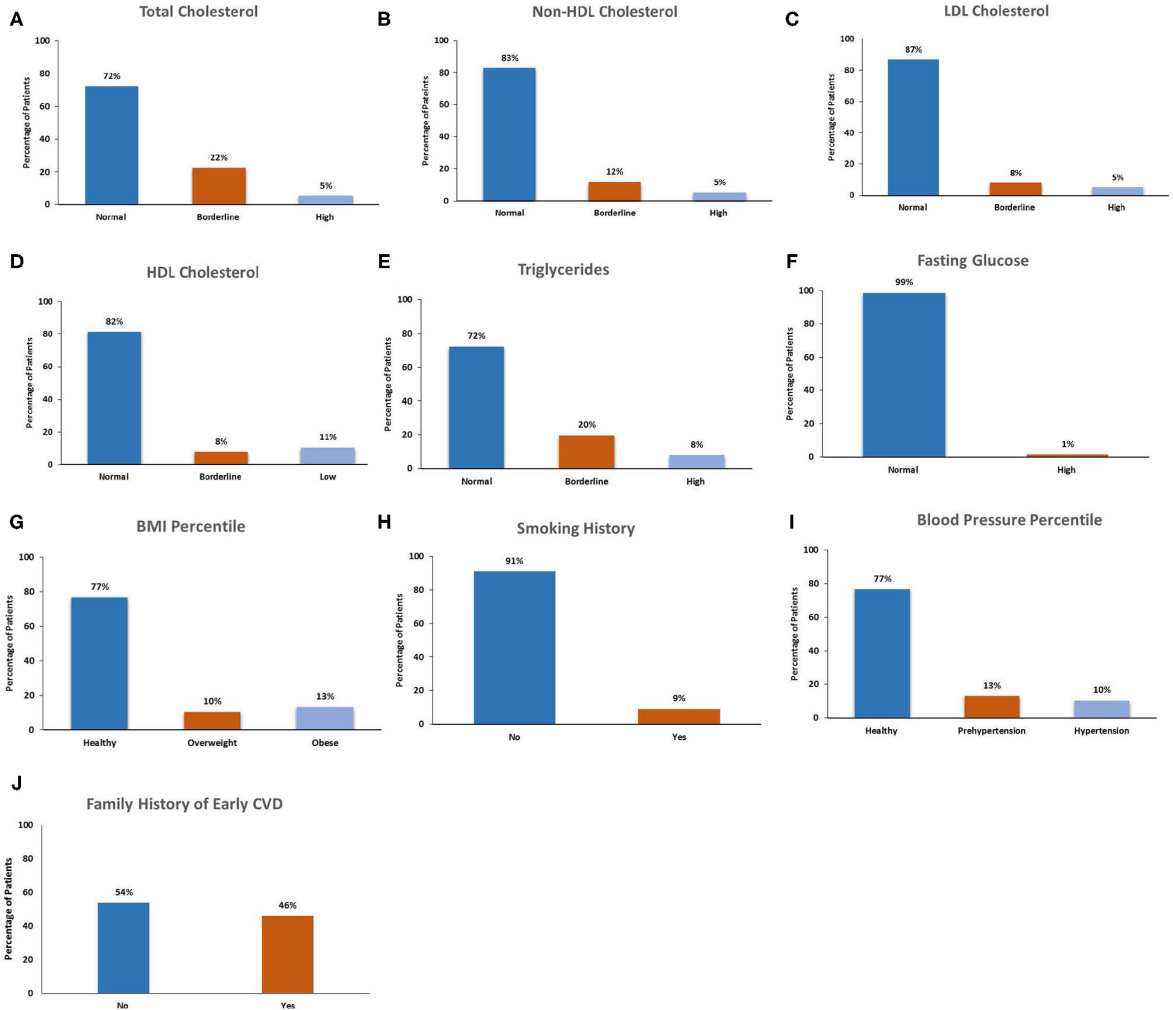
CVD risk factor categorization among children and adolescents with MDD.

The cardiovascular risk factors among participants are presented in Table 2 and Figure 1. Nearly half (46%) of participants had a family history of early CVD. The majority of participants (91%) were current non-smokers. On examination, 25% of participants had a BMI in either the overweight (11%) or obese (14%) ranges, and 25% of children had blood pressures in the pre-hypertensive (14%) or hypertensive (11%) ranges. On laboratory investigation, total cholesterol levels were elevated among 28% of participants. Lipoprotein level met or exceeded high-risk thresholds among 18% of children for HDL cholesterol, 17% for non-HDL cholesterol, 13% for LDL cholesterol and 27% for triglyceride concentrations. Among total cholesterol, non-HDL cholesterol, LDL cholesterol and TGs, a greater number of participants had levels in the “at-risk” compared with the “high risk” range. In contrast, more participants exhibited HDL levels in “high risk” compared with the “at-risk” range.

**Table 2:** Cardiometabolic risk factors among adolescents with MDD.

	**Mean (SD)**	***N***
Body mass index (kg/m^2^)	21.7 (5.3)	77
BMI percentile^*^	57.2 (29.5)	76
Systolic blood pressure (mmHg)	111.4 (13.7)	77
Diastolic blood pressure (mmHg)	63.3 (7.5)	77
Blood pressure percentile^*^	59.9 (18.1)	77
Fasting glucose (mmol/L)	5.0 (0.3)	76
Lipids		76
Total cholesterol (mmol/L)	4.0 (0.8)	
Non-HDL cholesterol (mmol/L)	2.5 (0.7)	
LDL cholesterol (mmol/L)	2.1 (0.7)	
HDL cholesterol (mmol/L)	1.4 (0.3)	
Triglyceride (mmol/L)	1.0 (0.6) 2.0	

* *Based on standardized norms for age and sex. BMI, body mass index; HDL, high density lipoproteins; LDL, low density lipoproteins*.

Comparison of CVD risk factors among healthy and overweight/obese participants are presented in Table 3. Overweight/obese participants had increased total cholesterol concentrations compared with healthy-weight participants (47 vs. 21%, *p* = 0.02). Of cholesterol components, non-HDL cholesterol concentrations were increased in overweight/obese participants compared with those of healthy weight. There were no significant differences in the proportion of participants with other CVD risk factors, including HDL cholesterol concentration, plasma glucose concentration, cigarette smoking, and family history of early CVD, between overweight/obese and healthy weight groups.

**Table 3:** CVD risk factors among healthy-weight vs. overweight/obese children and adolescents with MDD.

	**Healthy weight** **(*n* = 58)**	**Overweight/** **obese** **(*n* = 19)**	**Statistic**
	**Number (%)**	**Number (%)**	***X^**2**^, P-*value**
FH of Early CVD	22 (38)	10 (53)	1.27, *p* = 0.26
Abnormal FLP (any)	33 (57)	13 (68)	0.79, *p* = 0.37
Total cholesterol	12 (21)	9 (47)	5.14, ***p*** **=** **0.02**
Non-HDL cholesterol	6 (10)	7 (37)	7.36, ***p*** **=** **0.01**
HDL cholesterol	11 (19)	3 (16)	0.10, *p* = 0.76
LDL cholesterol	6 (10)	4 (21)	1.31, *p* = 0.25
Triglycerides	13 (22)	8 (42)	2.80, *p* = 0.09
Smoking history	6 (10)	1 (0.1)	0.45, *p* = 0.50
BP	11 (19)	7 (37)	2.55, *p* = 0.11
Fasting glucose	1 (0.02)	0 (0.0)	N/A

*FH, family history; CVD, cardiovascular disease; FLP, fasting lipid profile; HDL, high density lipoprotein; LDL, low density lipoprotein; BP, blood pressure. Bold values indicate that the chi-square test was statistically significant at the 0.05 level*.

### Cumulative Number of CVD Risk Factors

**Figure 2:**
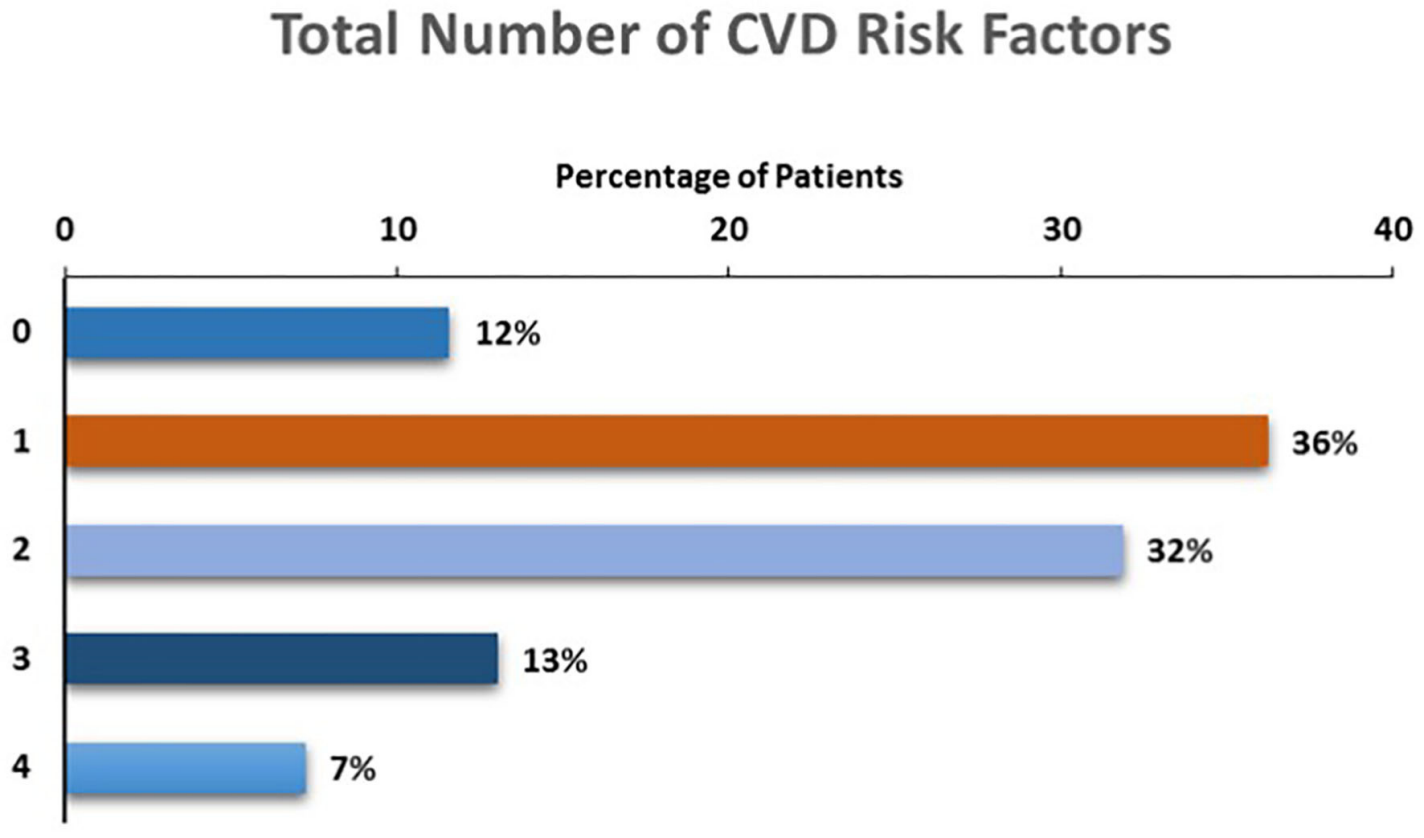
Number of CVD risk factors among children and adolescents with MDD.

The total number of CVD risk factors present among children with depression is presented in Figure 2. The majority (88%) of participants had at least one risk factor, with more than half (52%) of participants demonstrating two (32%), three (13%) or four (7%) risk factors for future CVD.

## Discussion

This study finds that objective markers of increased CVD risk are common among children and adolescents with depression. Over half of all participants had two or more CVD risk factors, even though 75% of children were in the healthy weight range. A significant proportion (46%) of children with depression had a family history of early CVD.

This study also finds that approximately one quarter of children with MDD have adverse lipoprotein and/or triglyceride patterns within clinically significant ranges which cannot be wholly attributed to overweight or obesity. Similarly, approximately one-quarter of participants had blood pressures in the pre-hypertensive or hypertensive range. Participants' mean cholesterol, BP and fasting glucose values in this study are comparable to those reported in the only other study of physiological risk factors among adolescents with MDD of which we are aware (11). In regard to obesity, these data are consistent with those reported by Merikangas et al. among 4,150 adolescents in the National Health and Nutritional Examination Survey, in which MDD was not associated with obesity after controlling for demographic variables (22).

Nearly 20% of participants in this study demonstrated significantly decreased HDL cholesterol concentrations, considered to be a protective factor for future CVD. This figure is in keeping with studies of adults with MDD, with some (but not all) studies reporting decreased HDL cholesterol only among adults with longstanding MDD (23–25). Also similar to research among adults with MDD, a significant proportion of adolescents with MDD in our study exhibited increased LDL cholesterol (25, 26). Together with the high number of children from families with increased early CVD, our findings thus suggest that early-onset of MDD may indicate a severe subtype of illness with respect to future CVD. Moreover, given extant literature confirming increased inflammation in MDD (27), anti-inflammatory effects of HDL cholesterol, and inflammation-driven elevations in LDL cholesterol (28), our findings are also consistent with theories suggesting that inflammation may be a mediator of the relationship between MDD and CVD, and that these effects may be exerted via circulating lipoprotein levels.

Despite their young age, the majority of participants with depression in this study were found to have at least two CVD risk factors, while three (or more) CVD risk factors were present in one in five depressed youth. These data compare with those of Waloszek et al. (11) among 17 predominantly female adolescents (mean age 16 years) with MDD, in which 59% of participants were categorized as being of moderate or high risk for future CVD based on a calculated risk score. Our findings are also in contrast to one study examining CVD risk factors in healthy children, in which 42 out of 214 (19.6%) 7 to 15 year old children demonstrated two or more CVD risk factors (29). The current study further extends those of Waloszek et al. (11) by examining a cluster of risk factors determined by history, physical examination and laboratory examination in a larger, younger sample of depressed children and adolescents and assessing these parameters in relation to weight status and clinical monitoring guidelines. Our results also align with the conclusions of the recent scientific statement from the American Heart Association Atherosclerosis, Hypertension and Obesity in Youth committee, advocating for inclusion of mood disorders such as MDD as Tier 2 (moderate) risk conditions (8). Indeed, were this designation to occur, the majority of participants in the current study would subsequently become identified as belonging to a Tier 1 (high) risk level by clinicians following the relevant NIH algorithm, given the burden of CVD risk factors we observed.

Nearly half of the children and adolescents with MDD in our study had family histories of early CVD. These findings are in keeping with the only previous study of this topic of which we are aware; in a study of 210 families of participants with child-onset depression, Rottenberg et al. found up to a four-fold increase in early CVD among families of MDD youth compared with control families (ORs 1.62–4.36, CIs = 1.03–15.40) (30). Taken together, the findings of the present study thus add to those suggesting that shared vulnerabilities may underlie the MDD-CVD association.

Both MDD and CVD are heritable disorders, with the proportion of the variance due to genetic factors estimated at 37–40% and 30–60%, respectively (31, 32). With respect to MDD, however, research aimed at identifying specific, possibly causal genetic variants has not been fruitful (33). Studies examining potentially common genetic factors that may predispose individuals to both MDD and CVD have suggested that shared genetic pathways way belie the relationship between MDD and coronary microvascular dysfunction (34) and between MDD and decreased heart rate variability among middle-aged male veteran twins (35). The results of the present study add to those above, finding that among the predominantly female study sample, families of children with MDD also demonstrate increased early-onset CVD. This suggests the possibility of identifying a more homogeneous subgroup of individuals with depression at risk for poor CVD outcomes for further genetic investigation.

The current study possesses many strengths, including (1) use of gold standard methods for determining psychiatric diagnoses, a semi-structured diagnostic interview; (2) objective measurement of a combination of cardiovascular risk factors including physiologic factors; and (3) assessment of child- and adolescent-aged participants who are early in the course of MDD, and do not have the chronic comorbidities (e.g., type 2 diabetes, substance use disorders) or overt manifestations of CVD that are common among adults and obscure the examination of the MDD-CVD association. Despite these strengths, however, this study has limitations. First, the absence of a control group limits our ability to compare these data to those of healthy children and adolescents, and prevents calculation of the magnitude of added CVD risk conferred by the presence of MDD. Studies of traditional CVD risk factors among children and adolescents who have been rigorously assessed for the absence of MDD, however, are few. Compared with published data of physically healthy children and adolescents (29), and with those of adolescents in which the absence of MDD has been established (11), our study finds a more than two-fold increase in the proportion of children at increased risk of future CVD based on cumulative CVD risk factor assessment (20 vs. 24 vs. 54%, respectively). A second study investigating family and lifestyle risk factors for CVD among adolescents with and without MDD in Hungary reported that 11% of non-MDD youth (mean age 15.8 years) were overweight or obese (30). The study investigators found that 22% of MDD youth were overweight or obese, similar to the proportion that we report (24%) in the current study. As examinations of CVD risk factors among children in national datasets do not indicate the presence or absence of MDD, comparison of our data with those of community-based surveys cannot be reliably made. Second, this study is of a clinical sample of children and adolescents with MDD who are help-seeking, as and such limits generalizeability of our results to non-clinical samples. The impact of this potential source of selection bias, however, is uncertain as our findings are comparable to those of Waloszek et al. (11) with respect to the proportion of adolescents at increased CVD risk in a community-based sample. While it is possible that children in this study were of increased MDD severity by virtue of their clinical presentation, population-based studies examining the severity of depression in non-treatment seeking community samples suggest that clinical presentation is not a reliable indicator of symptom burden (36). Finally, although the sample size of the present study is the largest of its kind among MDD youth, it is small in comparison to population-based samples. As such, the sample size limits the ability to detect small- to medium- effect sizes and adjust for numerous potentially relevant covariates in subgroup analyses. Rather, these data provide support for the need for future research among larger clinical samples of MDD youth to examine these questions.

## Conclusions

CVD risk factors are prevalent among children and adolescents with MDD, including those of a healthy weight. This study also finds a high rate of early CVD among family members of children and adolescents with depression. These findings suggest that routine cardiovascular risk factor screening may be warranted among youth with MDD, regardless of weight status, and may provide a valuable opportunity for prevention of future CVD.

## Data Availability Statement

The data that support the findings of this study are available from the corresponding author upon reasonable request.

## Ethics Statement

The studies involving human participants were reviewed and approved by Research Ethics Board, Hospital for Sick Children. Written informed consent to participate in this study was provided by the participants' legal guardian/next of kin.

## Author Contributions

DK, KC, CB, FM, and BM contributed to the conception or design of the work. DK, TP, CB, and BM contributed to the acquisition, analysis, or interpretation of data for the work. DK drafted the manuscript. All authors critically revised the manuscript and gave final approval and agree to be accountable for all aspects of work ensuring integrity and accuracy.

## Conflict of Interest

The authors declare that the research was conducted in the absence of any commercial or financial relationships that could be construed as a potential conflict of interest.

## Publisher's Note

All claims expressed in this article are solely those of the authors and do not necessarily represent those of their affiliated organizations, or those of the publisher, the editors and the reviewers. Any product that may be evaluated in this article, or claim that may be made by its manufacturer, is not guaranteed or endorsed by the publisher.
